# Transcriptomic in silico analysis of bovine *Escherichia coli* mastitis highlights its immune-related expressed genes as an effective biomarker

**DOI:** 10.1186/s43141-021-00235-x

**Published:** 2021-10-12

**Authors:** Farmanullah Farmanullah, Xianwei Liang, Faheem Ahmed Khan, Mohammad Salim, Zia ur Rehman, Momen Khan, Hira Sajjad Talpur, N. M. Schreurs, Mostafa Gouda, Sami Ullah Khan, Zhang Shujun

**Affiliations:** 1 Key Laboratory of Agricultural Animal Genetics, Breeding and Reproduction, Education Ministry of China, College of Animal Sciences and Technology, Huazhong Agricultural University, Wuhan, 430070 People’s Republic of China; 2 Faculty of Veterinary and Animal Sciences, National Center for Livestock Breeding Genetics and Genomics LUAWMS, Uthal, Pakistan; 3 Key Laboratory of Buffalo Genetics, Breeding and Reproduction technology, Ministry of Agriculture, Buffalo Research Institute, Chinese Academy of Agricultural Sciences, Nanning, 530001 China; 4 Department of Forestry and Wildlife Management, University of Haripur, Haripur, Khyber Pakhtunkhwa Pakistan; 5 Department of Animal Health, Faculty of Animal Husbandry and Veterinary Sciences, UAP, Peshawar, Pakistan; 6 Livestock and Dairy Development, Peshawar, Khyber Pakhtunkhwa Pakistan; 7 Animal Science, School of Agriculture and Environment, Massey University, Private Bag 11222, Palmerston North, New Zealand; 8 College of Biosystems Engineering and Food Science, Zhejiang University, Hangzhou, 310058 China; 9 Department of Nutrition and Food Science, National Research Centre, Giza, 12622 Egypt; 10 Department of Internal Medicine, Faculty of Veterinary Medicine, Universitas Gadjah Mada, Yogyakarta, Indonesia

**Keywords:** Mastitis, Escherichia coli, Microarray, Transcriptomic analysis, Dairy cow, Differentially expressed genes

## Abstract

**Background:**

Mastitis is one of the major diseases causing economic loss to the dairy industry by reducing the quantity and quality of milk. Thus, the objective of this scientific study was to find new biomarkers based on genes for the early prediction before its severity.

**Methods:**

In the present study, advanced bioinformatics including hierarchical clustering, enrichment analysis, active site prediction, epigenetic analysis, functional domain identification, and protein docking were used to analyze the important genes that could be utilized as biomarkers and therapeutic targets for mastitis.

**Results:**

Four differentially expressed genes (DEGs) were identified in different regions of the mammary gland (teat cistern, gland cistern, lobuloalveolar, and Furstenberg’s rosette) that resulted in 453, 597, 577, and 636 DEG, respectively. Also, 101 overlapped genes were found by comparing 27 different expressed genes. These genes were associated with eight immune response pathways including NOD-like receptor signaling pathway (IL8, IL18, IL1B, PYDC1) and chemokine signaling pathway (PTK2, IL8, NCF1, CCR1, HCK). Meanwhile, 241 protein-protein interaction networks were developed among overlapped genes. Fifty-seven regulatory events were found between miRNAs, expressed genes, and the transcription factors (TFs) through micro-RNA and transcription factors (miRNA-DEG-TF) regulatory network. The 3D structure docking model of the expressed genes proteins identified their active sites and the binding ligands that could help in choosing the appropriate feed or treatment for affected animals.

**Conclusions:**

The novelty of the distinguished DEG and their pathways in this study is that they can precisely improve the detection biomarkers and treatments techniques of cows’ *Escherichia coli* mastitis disease due to their high affinity with the target site of the mammary gland before appearing the symptoms.

**Graphical abstract:**

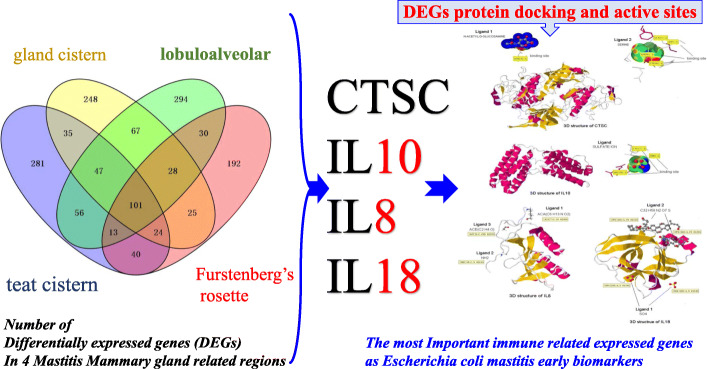

**Supplementary Information:**

The online version contains supplementary material available at 10.1186/s43141-021-00235-x.

## Background

Mastitis disease is a parenchymal inflammation of the dairy cattle mammary gland that is one of the most expensive problems in global milk production [1]. In which, mastitis caused significant economic losses for the global dairy industry [2]. The predisposing factors and type of pathogen are important and have a crucial role in the severity and outcome of the complex and multi-etiological nature of the disease, whereas combination of pathogen, environment, host, location, and disease severity makes it more complex and costly [3].

The potential use of bioinformatics analysis for early detection and exploring the pathway’s mechanisms of mastitis infection and its related genes has become one of the hot areas in this field [4]. In which, the advanced bioinformatics models predicted what may happen on the basis of mastitis or even other diseases inflammation. And the most adapted treatment is based on the degree of severity that facilitates early recovery, avoids a significant reduction in reproductive performance, and helps to prevent transmission to other cows. However, little is known about gene articulation in the mammary gland during the infection [5]. Microarray and quantitative real-time PCR techniques have been used to identify genes that become activated in various regions of the mammary gland during the acute period of experimentally induced *Escherichia coli* mastitis [6, 7]. *E*. *coli* infection in mammary tissue shows an increase in TLR2 and TLR4 expression [8]. Mediator and inflammatory cytokine expression are activated by Toll-like receptors (TLRs) which are implicated in cell differentiation, apoptosis, and the immune response. Additionally, the immune response and cytokine’s role in the spread of mastitis has been reported by several literatures [9]. The pro-inflammatory cytokines increase locally and systemically in *E*. *coli-*infected glands and mediate the host response via TNF-alpha, IL8, and interleukin 1 beta (IL1B) expression. The transforming growth factor beta induced (TGFBI) and IL-10 cytokines are upregulated during *E*. *coli* mastitis, as anti-inflammatory cytokines, and persist for the period of the inflammatory response [10]. A total of 881 differentially expressed genes (DEG) (605 unregulated and 276 downregulated) were distinguished in mammary glands of lactating Chinese Holstein dairy cows compared to dry cows. These genes are responsible for transcription and protein modifications that correlated to mammary gland cell proliferation, cycle, apoptosis, and other immune-related pathways. For instance, RNA-sequencing (RNA-Seq) of the bovine mammary organ at peak lactation of small-tailed sheep (STH) and Gansu Alpine Merino (GAM) provided 74 transcriptome genes profiles that were higher expressed in STH compared with GAM breed. Likewise, 143 genes that were expressed at lower levels in STH compared with GAM were identified. In which, transcriptomic databases, like gene ontogeny (GO) and Kyoto Encyclopedia of Genes and Genomes (KEGG), revealed that these DEGs were related to the binding and catalytic activities, hematopoietic cell lineages, oxytocin signaling pathway, and neuroactive ligand-receptor interaction [11].

Furthermore, aggregates of 1103, 1356, and 1397 genes were differentially expressed between Kashmiri and Jersey cattle for multicellular organismal processes and catalytic activities. And these genes were responsible for the majority of the pathways involved in milk production of Jersey dairy cattle, like JAK (Janus tyrosine kinase)-STAT (signal transducer and activator of tranion) (JAK-STAT), p38 MAPK, and the PI3 kinase pathways [11]. A total of 3746 differentially expressed long non-coding RNAs (lncRNAs) (DELs) and 2890 DEGs were distinguished between the dry and lactating mammary glands of Holstein cows. For instance, the functional enrichment analysis on target genes of lncRNAs demonstrated that these genes were involved with lactation-related signaling pathways, including cell cycle, cell adhesion, and phosphatidylinositol-3-kinase (PI3K-Akt) signaling pathways. Furthermore, the interactions among lncRNAs and their potential microRNAs (miRNAs) were anticipated and mostly confirmed [12].

Arora et al. [13] analyzed the milk transcriptome from three lactation phases of Murrah buffalo by RNA sequencing. In that study, a total of 12,833 transcripts were consistent over all the stages and were associated with protein digestion, transport, and immune response. While 271, 205, and 418 were unique to early, mid, and late lactation respectively [13]. As a result, the correlation of the lactation’s various phases may lead to exploring novel genes or transcripts that manage the lactation process. Also, RNA sequencing offers a proficient and far-reaching depiction of the outflow of genes in a given tissue. This process is very important to understand and formulate a therapeutic protocol for bovine mastitis. Therefore, auxiliary research, investigation, and further insights in this area are needed to advance the treatment and disease control in dairy animals. Additionally, various analytical studies have explained the role that *E*. *coli* plays in mastitis to elucidate the potential mechanism by which *E*. *coli* impairs reproductive performance caused by mastitis in dairy animals. Therefore, specific and sensitive biomarkers for early detection of mastitis to allow early treatment need to be identified and the molecular mechanisms underlying *E*. *coli* mastitis need to be better understood.

Vincent et al. [10] mentioned that the local immunization response of dairy cows to *E*. *coli* mastitis vaccines has shown limited efficacy because of the modification of the cytokine profile (such as IFN-γ increase and TNF-α reduction). That study results support the idea that the mammary gland cell-mediated immune genes with DEG study links are playing a key role in understanding the *E*. *coli* vaccine-induced protection of the mammary gland. Also, in dairy animals infected with coagulated-positive *Staphylococci*, being in first or second lactation. Several 1700 DEGs were distinguished. The gene ontology functional characterization indicated that the molecular elements of DEGs were overrepresented in the action of cytokines, chemokines, and their receptors. In which, these DEGs are involved in molecular processes, like protein-calcium-lipid binding ability, chemokine activity, and protein homodimerization. Furthermore, gene network analysis has demonstrated that DEGs are related to inflammation, cell relocation, and immune reaction to diseases. Also, they could be responsible for the advancement of cells and tissues, and the cows’ humoral reactions [14].

Thus, this study aimed to find and analyze the insights into the *E*. *coli* common pathogenetic factors active in bovine mastitis, and to emphasize the genetic difference among those responses in the mammary gland. Microarray analysis was established to characterize the gene expression patterns and to process their networks that become activated in the bovine mammary regions during the acute phase of experimentally induced intramammary infection with *E*. *coli*. The knowledge of the present study could manage the drug discovery related to bovine mastitis through bioinformatics tools assistance for reducing the time and complications involved in the clinical trials related to mastitis disease. In which, the genome-wide analysis of miRNA profile’s relationship with DEG and their target protein active sites provided insight into bovine mastitis and inflammatory diseases in general.

## Methods

### Data set and description

The profiles of gene expression were downloaded from the Gene expression Omnibus (http://www.ncbi.nlm.nih.gov/geo/) with the accession number GSE15441 (platform: GPL3301 USDA Bovine 60mer 344k Array (gene layout) [6]. Three healthy lactating Holstein cows were used to extract four different regions of mammary gland tissues. All the mammary glands were free of any infections. Immediately, following milking in the morning, a 4 ml *E*. *coli* suspension (unit of hundred forming colony/ml) was infused into 1 mammary gland of the udder. The contralateral gland was infused with PBS (control gland). After 12 h, the remaining ipsilateral quarter was infused with *E*. *coli*. Cows were euthanized for 24 h after the initial infusions and tissues were collected from 4 regions of each mammary gland (Furstenberg’s rosette, cistern, lobuloalveolar, and teat cistern). Twelve samples were also analyzed from the same 4 different regions of mammary glands of 3 Holstein cows from infected and control mammary glands. A total of 24 samples were infused PBS or *E*. *coli* after 24 h for the follow-up analysis. Gene expression dataset GSE15020 [15] in gene expression omnibus (GEO), which also contains the mammary gland at 24 h following *E*. *coli* infusion in uninfected healthy mammary glands tissue, was used for validation. The data in GSE15020 were produced by the platform of [GPL2112] Affymetrix Bovine Genome Array. A total of 10 samples with two conditions: *E*. *coli* treated for 24 h vs. healthy control (five replicates for each condition) were included in this dataset.

### Data pre-processing and differentially expressed genes screening

The raw expression data files and the annotation files were downloaded, and the gene expression data of all samples were preprocessed and normalized by using background correction, quantile normalization, and probe summarization using linear models for microarray data (LIMMA) package of Bioconductor (http://www.bioconductor.org/packages/release/bioc/html/limma.html) [16]. The LIMMA package was also used to identify differentially expressed genes (DEGs) between control and infected samples at 24 h post-infection with *E*. *coli* from the four different regions under study. The same DEGs screening methods were used in the validation dataset, as well as the cutoff values.

### Hierarchical clustering and comparison analysis of selected DEGs in a different region

The expression of the selected DEGs in GSE15441 at 24 h post-infection with *E*. *coli* in the four different regions were used to generate hierarchical clustering image by using the P-heatmap package in R tools (RStudio, PBC, Boston, USA) [17]. Then overlapping DEGs (ODEGs) were identified in each group by drawing Venn diagrams using the Venn diagram package in RStudio (https://cran.r-project.org/web/packages/VennDiagram/).

### Enrichment analysis for the ODEGs in the four groups

To explore the functions of overlapping differentially expressed genes (ODEGs) and their pathways in *E*. *coli*-infected mammary samples, the Database for Annotation, Visualization and Integrated Discovery (DAVID; https://david.ncifcrf.gov/), GO, and KEGG were performed. The *P* value < 0.05 and gene count ≥ 2 were set as the cut-off criteria. Furthermore, the category of enriched GO, KEGG terms, and the gene number were displayed as a histogram which was constructed by ggplot2 package in R [18].

### Protein-protein interaction network analysis for ODEGs

STRING version 10.0 (http://string-db.org/) was used to predict the protein-protein interaction (PPI) relationships of overlapped DEGs (gene pairs with PPI score > 0.4 were used), and Cytoscape 3.2.0 (http://www.cytoscape.org/) were used to construct the PPI network figure.

### miRNA-DEGs-TF regulatory network analysis

- The miRTarBase database version 2016 (http://mirtarbase.mbc.nctu.edu.tw/) [19] was used to search for miRNAs that target DEGs in the PPI network. The miRNAs obtained from database searching were compared with those reported in the database of cattle candidate genes and genetic markers for milk production and mastitis to find bovine mastitis-related miRNAs.
- Searching related transcription factors (TFs) were performed through WebGestalt (http://www.webgestalt.org/option.php) database, which is a functional enrichment analysis tool that can predict the TFs which regulates the ODEGs in PPI network. *P* value < 0.05 was set as the significance cut-off criteria. Results of (a) and (b) were integrated and miRNA-DEGs-TF regulatory networks were constructed by Cytoscape 3.2.0 (http://www.cytoscape.org/).

### Active site prediction of interested DEGs

In biology, the active site is the region of an enzyme where substrate molecules bind and undergo a chemical reaction. Ligands and enzymes are usually bound to the active site of the protein. However, enzyme inhibitors may adhere to the active site and block it. This is very important for the design of enzyme inhibitors. Therefore, we searched active sites of DEGs of interest by using scanprosite (http://prosite.expasy.org/scanprosite/) (Table 1).

**Table 1:** The results of pathway enrichment analysis for the ODEGs

Pathway name	Count	***P*** value	Genes
**hsa04621: NOD-like receptor signaling pathway**	4	0.004551	IL8, IL18, IL1B, PYDC1
**hsa04062:Chemokine signaling pathway**	5	0.018165	PTK2, IL8, NCF1, CCR1, HCK
**hsa00520: Amino sugar and nucleotide sugar metabolism**	3	0.023987	HK3, UGDH, CHIT1
**hsa04142:Lysosome**	4	0.025594	SLC11A2, SLC11A1, CD68, CTSC
**hsa04670:Leukocyte trans endothelial migration**	4	0.026166	PTK2, NCF2, NCF1, ITGAM
**hsa04060:Cytokine-cytokine receptor interaction**	5	0.053251	IL8, CCR1, IL18, IL1B, IL10
**hsa04662:B cell receptor signaling pathway**	3	0.063407	FCGR2B, LILRB3, NFATC2
**hsa04666:Fc gamma R-mediated phagocytosis**	3	0.095449	FCGR2B, NCF1, HCK

### Epigenetic analysis, epigenetic modifications, and CpG island

In epigenetic mechanisms, gene nucleotide sequences of cathepsin C (CTSC), IL10, IL8, and IL18 were downloaded from the NCBI database complete with the promoter sequences (1000 bps upstream of the transcription start site), as listed in Table 2. Meth primer 2.0 (http://www.urogene.org/cgi-bin/methprimer/methprimer.cgi) software was used to predict CpG islands within the following parameters: CpG island length > 200 bp, GC content > 50%, observed/expected CpG ratio > 0.6.

**Table 2:** Gene location information list

Gene	Chromosome	Location		Promoter	CpG island
		Start	End		
**CTST**	29	7433445	7473316	Upstream-1000bp	1
**IL8**	6	90559882	90563647	Upstream-1000bp	None
**IL10**	16	4402474	4406412	Upstream-1000bp	
**IL18**	15	22800420	22826701	Upstream-1000bp	

### Functional domain identification and mutation analysis

Simple Modular Architecture Research Tool (SMART; http://smart.embl-heidelberg.de/) was used for functional domain identification (FDI). Also, the genomic variants database was used to find copy number variants (CNVs) and single-nucleotide polymorphisms (SNPs) of CTSC, IL10, IL8, and IL18 (http://dgv.tcag.ca/dgv/app/about?ref=GRCh37/hg19).

### Protein docking exploration of active immune response genes

The 3D structure of 4 selected proteins (CTSC, IL10, IL8, and IL18) that their ligands that play a key role in protein docking were analyzed. Also, protein sequence and structure characterization (α-helix, β-sheet) were performed. In brief, a molecular docking simulation between the key DEGs based on network analysis and the target proteins. Protein Data Bank (PDB, http://www.rcsb.org) was used for downloading the target proteins. The protein structure and active components were modified by AutoDockTools [20, 21]. The processed protein and DEGs were introduced into AutoDock Vina for molecular docking.

### Protein sequence-based voluntary

Protein sequence-based voluntary tree to show the homology with other sequences of different species and to find out the similarity of CTSC protein in various species protein sequence alignment was performed. The public database of NCBI (https://www.ncbi.nlm.nih.gov/ncbisearch/) was used to define the protein alignment, the protein sequence of 5 different species under study (*Bos taurus*, *Homo sapiens*, *Mus musculus*, *Canis lupus*, and *Sus scrofa*).

## Results

### Processing of data and screening of differentially expressed genes

**Fig. 1:**
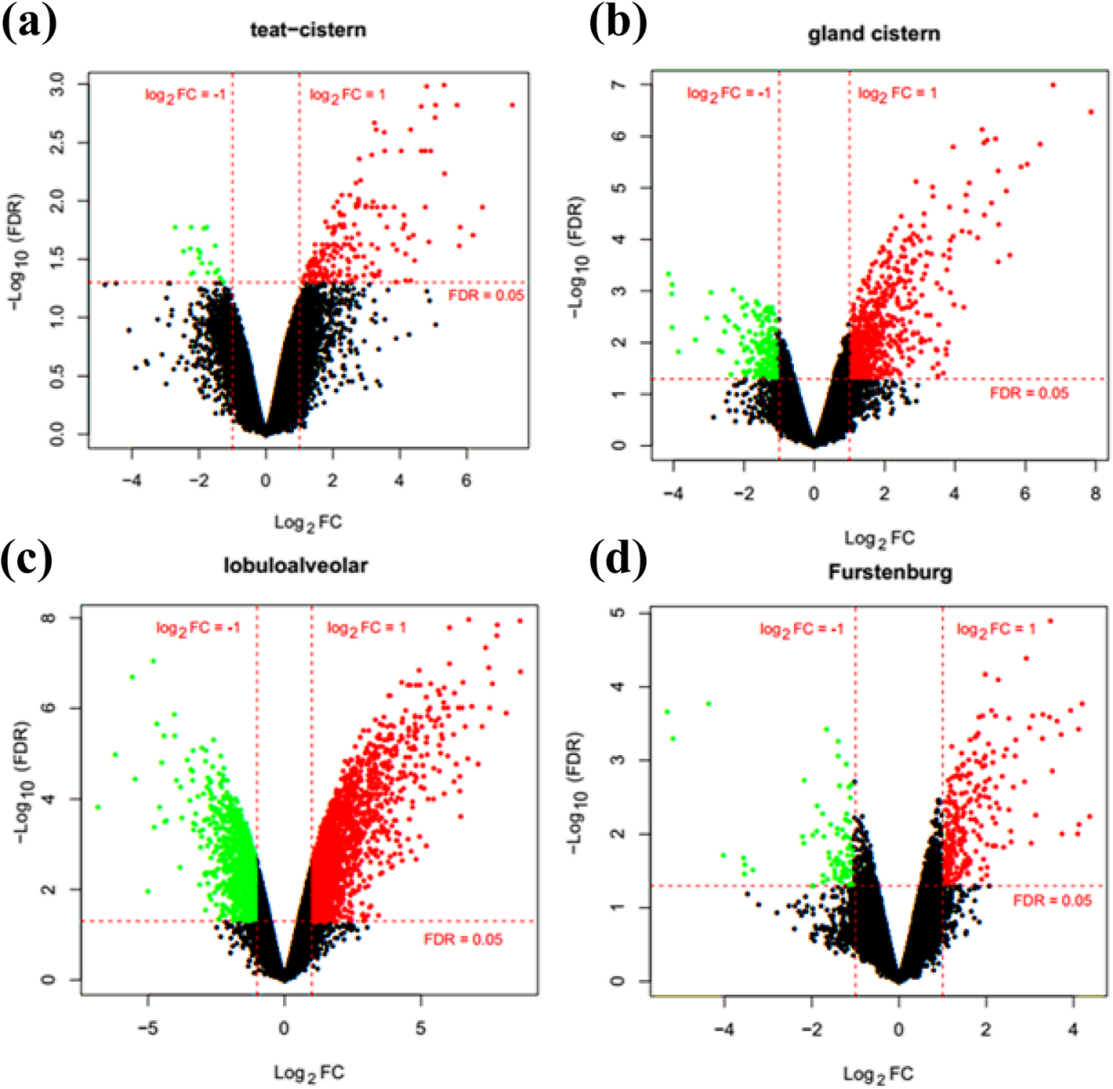
Volcano plot of DEGs in teat cistern (**a**), gland cistern (**b**), lobuloalveolar (**c**), and Furstenberg’s rosette (**d**), regions. Red horizontal dot line means FDR = 0.05 cutoff line, two red vertical dot lines mean logFC = 1 and logFC = − 1 cutoff line. Gene dots that were distributed above the FDR = 0.05 red line and out of |logFC| = 1 were the DEGs we identified

The data were normalized and the detailed normalized expression data can be found in Volcano plots for each region (Fig. 1). In which, only the genes meeting FDR < 0.05 and |log_2_ fold change (FC)| > 1 were chosen as DEGs. It could be observed that the normalization process controlled the variation between the population’s replicates (Fig S1). Based on the cut-off criteria, the DEGs of 453, 597, 577, and 636 were identified from the teat cistern (Fig. 1a), gland cistern (Fig. 1b), lobuloalveolar (Fig. 1c), and Furstenberg’s rosette (Fig. 1d) regions, respectively.

#### Differential Express Genes screening and data processing

**Fig. 2:**
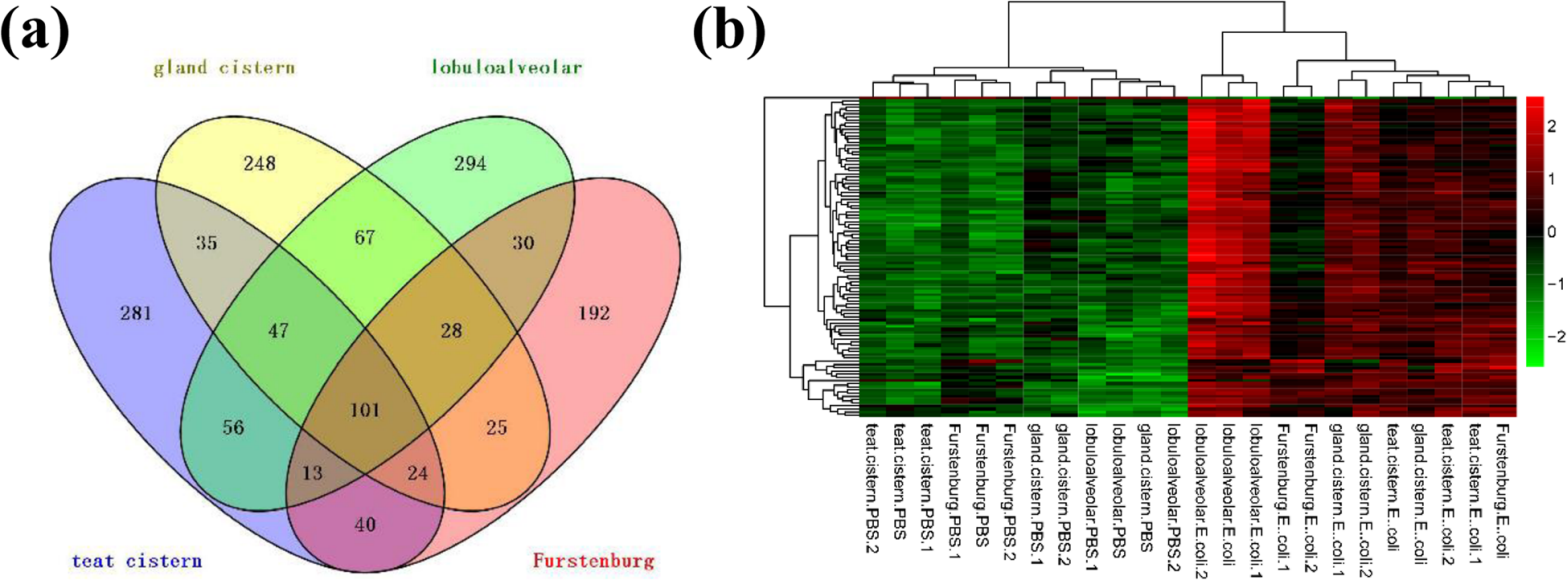
**a** Venn diagram of DEGs in the four different regions. **b** Hierarchical clustering of 101 overlapping differentially expressed genes (ODEGs)

The DEG expressions from the four different regions (teat cistern, gland cistern, lobuloalveolar, and Furstenberg’s rosette) were represented by hierarchical clustering heatmaps, as shown in Fig S2. In which, each group was divided into two types (*E*. *coli*-infected and control). In the teat cistern, it was observed that *E*. *coli*-infected was generally upregulated compared to the control group. However, in Furstenberg’s rosette, the changes in expression pattern between *E*. *coli*-infected and control samples were lower. Also, there were 101 overlapped DEGs (ODEG; 100 upregulated and 1 downregulated the gene) (Listed in Table S1). These ODEG were not affected by different tissue regions and were all associated with mastitis (Fig. 2a). Also, the 101 ODEGs had consistent expression patterns in different regions (Fig. 2b).

### Gene ontology and KEGG pathway analysis for the ODEGs

A total of 27 GO biological processes (BP) for the co-regulated DEGs were generated. In which, 23 of these genes are participating in the immune response, 19 genes with defense response, and 9 genes with inflammatory response. Also, several genes were directly expressed in response to the bacterium (like SLC11A1, IL1B, LBP, IL10) (Table S2). For instance, GO:0006955~immune response gene had the most gene count and FDR with 1.47 × 10^−12^ (Fig S3). According to KEGG enrichment analysis, ODEGs were significantly enriched in 8 pathways, such as NOD-like receptor signaling pathway (IL8, IL18, IL1B, PYDC1) and the chemokine signaling pathway (PTK2, IL8, NCF1, CCR1, HCK) (Table 1).

### Protein-protein interaction network analysis for ODEGs

The STRING database was used to search for PPI relationships among ODEG. In which, 241 PPI pairs were identified with a combined score over than 0.4. The PPI network was constructed, involving 55 upregulated ODEGs (Fig S4). A high significant direct network was found between IL1B and IL8. However, PTK2 was indirectly linked to IL8 through its link with IL1B.

### Transcription factor-miRNA-target regulatory network analysis

**Fig. 3:**
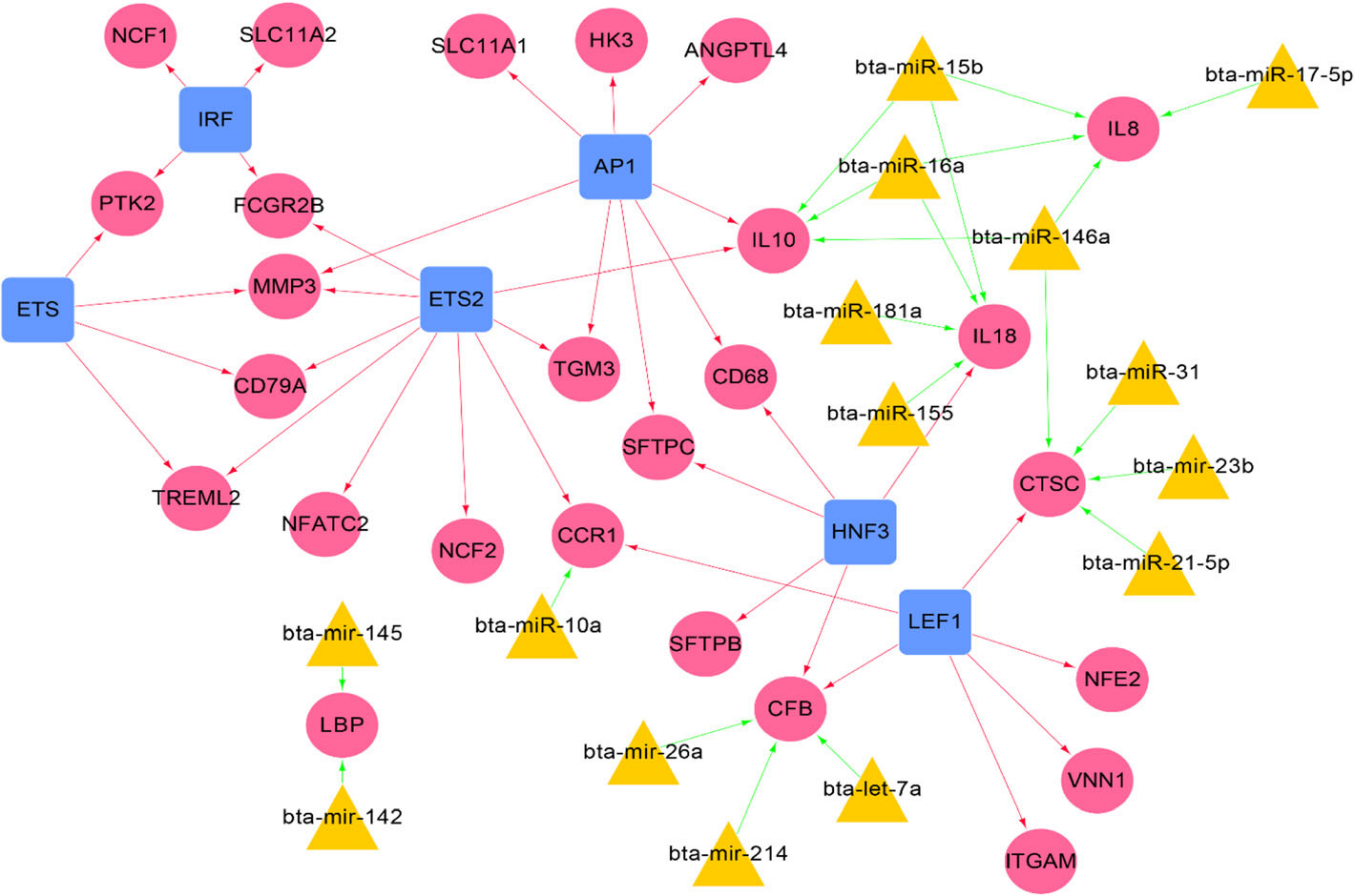
miRNA-DEG-TF regulatory network. Red rounded nodes represent DEGs, yellow trigonal nodes represent miRNAs, and blue quadrate nodes represent significantly related TFs; red arrow lines mean TF-DEGs regulatory relationships, green arrow lines mean miRNA-DEGs regulatory relationships

In total, 57 regulatory relationships between miRNAs, transcription factors (TFs), and ODEGs that had PPI relationships were identified to construct the miRNA-DEG-TF regulatory network. The miRNA-DEG-TF regulatory network contained 21 miRNA-DEG pairs and 36 TF-DEG pairs of s regulatory relationships, including 16 miRNAs obtained from miRTarBase database (Table S3) and 6 significantly enriched TFs obtained from WebGestalt (Table S4). In which, AP1 FDR was calculated 3.59 × 10^−5^ for the 9 counts of target DEGs: CD79A, FCGR2B, CCR1, MMP3, TREML2, NFATC2, IL10, NCF2, TGM3 (Table S4). Also, two parts of the regulatory relationships were integrated to construct the miRNA-DEG-TF regulatory network. For instance, IL18-DEG was linked with four microRNAs (bta-miR-15b, 16a, 181a, and 155); also, it was integrated with the HNF3-TF (Fig. 3).

Meanwhile, 5 miRNAs were related to bovine mastitis inflammation from the databases of cattle candidate genes and genetic markers for milk production and mastitis. These miRNAs included bta-miR-146a, bta-miR-21-5p, bta-miR-31, bta-miR-16a, and bta-miR-155. There were 4 DEG that were targeted by the bovine mastitis inflammation-related miRNAs. CTSC was regulated by bta-miR-146a, bta-miR-21-5p, bta-miR-31, as well as was modulated by 3 TF (LEF1, IL10, and IL8). Also, bta-miR-146a and bta-miR-16a, and IL 10 were regulated by 2 transcription factors (LEF1 and AP1). While IL18 was regulated by bta-miR-16a and bta-miR-155, and by HNF3 transcription factor. As a result, the 4 mentioned DEGs that are regulated by bovine mastitis inflammation and that was correlated with miRNAs and TFs were selected for further analysis.

### Validation of the expression level of DEGs

**Fig. 4:**
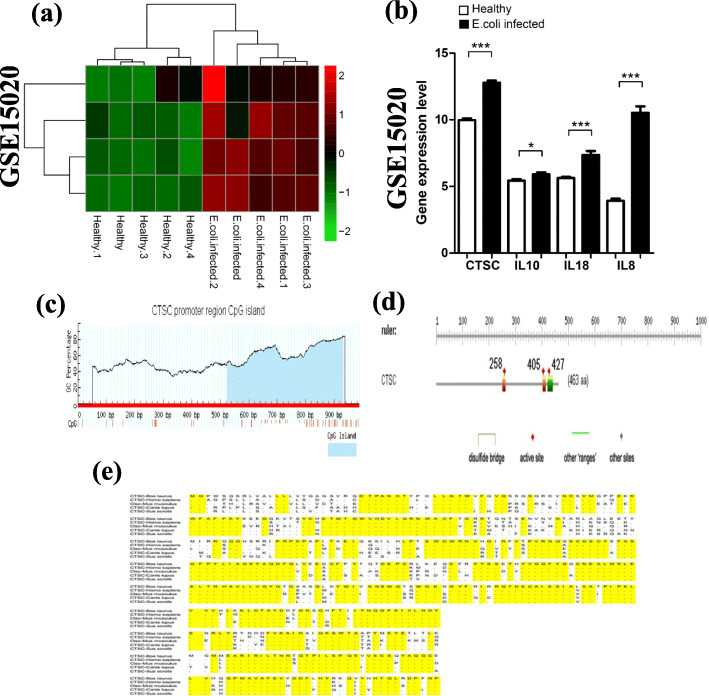
**a** Heatmap of selected 4 DEGs. **b** Barplot of selected DEGs. * Refers to 0.01 ≤ *p* < 0.05, ** refers to 0.005 ≤ *p* < 0.01, *** refers to *p* < 0.005. **c** CpG island in the promoter region of CTST. The blue-filled region is CpG Island. **d** Presents the active sites in the differentially expressed gene (DEG) of CTSC. **e** Alignment of CTSC protein among 5 different species. The highlight sequence parts with yellow were consensus sequences of different species

In the studied dataset, GSE15441, CTSC, IL10, IL8, and IL18 were upregulated. To validate the expression of the selected 4 DEGs in the GSE15441 dataset, another dataset GSE15020 was used (Fig. 4a). In addition, CTSC, IL10, IL8, and IL18 were all expressed at higher levels (*P* < 0.05) in *E*. *coli*-infected samples than that in healthy samples (Fig. 4b). In which, the expression of IL8 had the highest increase (*P* < 0.001) compared to IL10. Also, the expression patterns in GSE15020 were the same as GSE15441; therefore, all the 4 selected DEGs (CTSC, IL10, IL8, and IL18) could be validated in GSE15020.

### Epigenetic analysis, epigenetic modifications, and CpG island and active cite

Methylation is one of the most important epigenetic modifications. This process includes the addition of methyl groups to the DNA, mostly at CpG sites, to convert cytosine to 5-methylcytosine. As a result, these highly methylated areas tend to be less transcriptionally active. CpG islands were predicted which are the main areas of methylation for the interested genes (gene nucleotide sequences of CTSC, IL10, IL8, and IL18) based on genomic sequences. In this study, CpG islands were found in the promoter region in CTST (from about 500 bp to 900 bp upstream of transcription start site), while no CpG islands were found in IL10, IL8, and IL18 (Fig. 4c). In which, 3 active sites in CTSC were found (Fig. 4d). These 3 active sites were distributed at the 258 aa, 405 aa, and 527 aa positions.

### Functional domain identification

We used Simple Modular Architecture Research Tool (SMART, http://smart.embl-heidelberg.de/), which allows the identification and annotation of genetically mobile domains and the analysis of domain architectures. More than 500 domain families found in signaling, extracellular, and chromatin-associated proteins were detected. The results of confidently predicted domains in CTSC, IL10, IL8, and IL18 were listed in Table 3. In which, IL10 domain residues started from 38 to 174; however, CTSC started from 231 to 458. In which, protein sequences alignments from the 5 different species (Bos Taurus, Homo sapiens, Mus musculus, Canis lupus, and Sus scrofa) were compared and highlighted with yellow color to find the consensus sequences of CTSC protein (Fig. 4e). Also, a very intersecting upstream SNP (rs104894210) has been found in CTSC, and two upstream SNPs were in IL8 (rs1000003014, rs1000275946). On the other hand, IL18 had no upstream SNPs (Table S6). In addition, Table S5 listed the CNVs of each gene from the database of Genomic Variants (DGV). In which, novel sequence insertion was in CTSC and two deletion subtypes (nsv1134175, esv2745077) were in IL18.

**Table 3:** Confidently predicted domains in CTSC, IL10, IL8, and IL18

Domain Name	Start	End	***E*** value
**CTSC (Pept_C1)**	231	458	3.27E-81
**IL-8 (SCY)**	31	92	2.86E-23
**IL18**	51	190	2.01E-21
**IL10**	38	174	8.91E-107

### Protein docking exploration of vibrant immune response genes and the active site prediction of interested DEGs

**Fig. 5:**
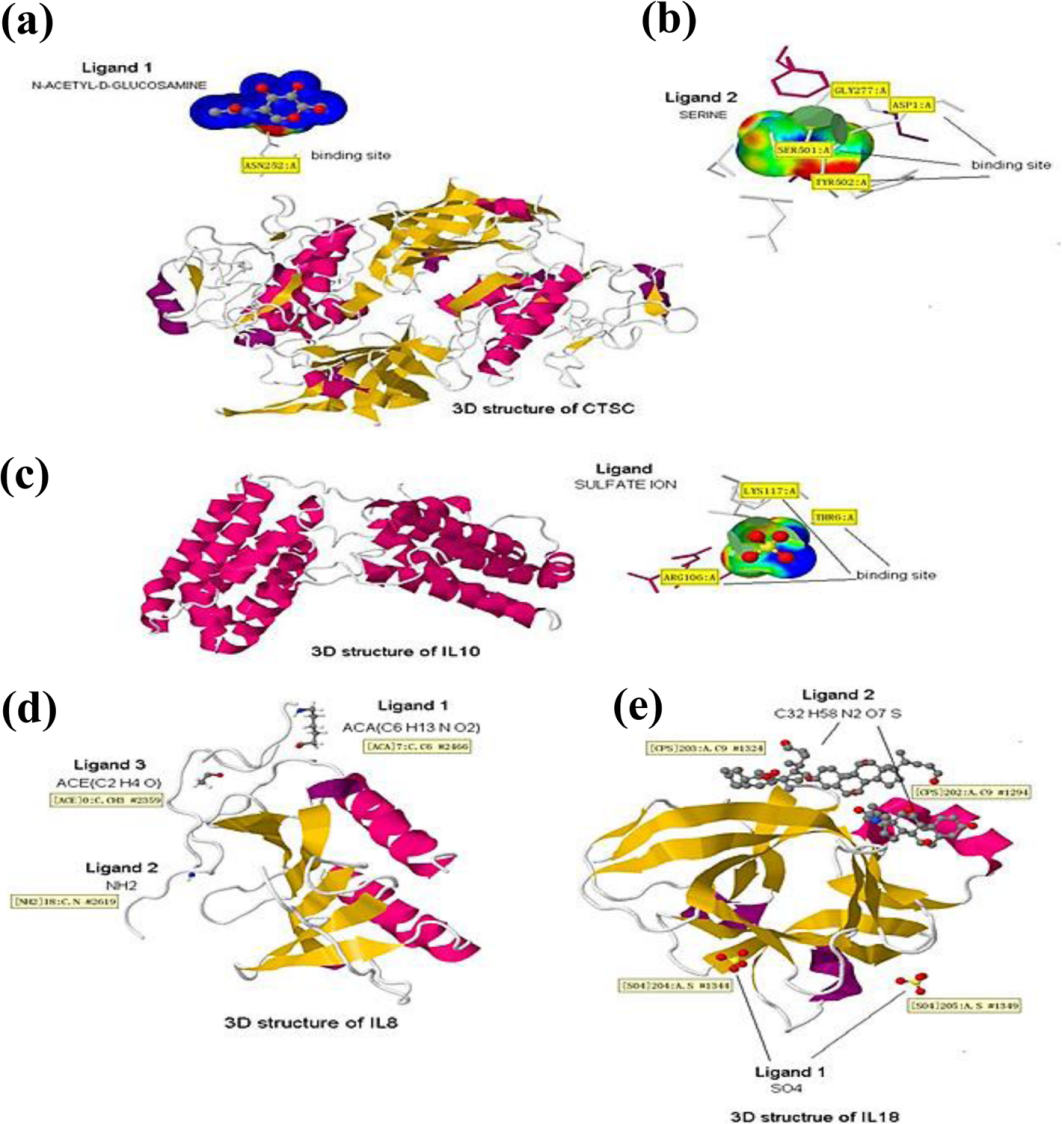
3D structure of four selected proteins. Ligand 1 and 2 were ligand molecular which can dock and bind to the active sites of proteins (**a**, **b**). Words in the yellow boxes were the information about the active binding sites. In the 3D structures, red ones mean α-helix, the orange ones mean β-fold. **a**–**e** Represents CTSC, IL10, IL8, and IL18

The 3D structure of the 4 selected proteins (CTSC, IL10, IL8, and IL18) was elucidated and the ligands for docking through protein binding active sites identity are presented in Fig. 5. The active sites of CTSC, IL10, IL8, and IL18 were identified using scanprosite. In which, 3 active sites in CTSC were found by the molecular docking (Fig. 5a, b), but no active sites in IL10, IL8, and IL18 (Fig. 5c, d, e). These 3 active sites were distributed at the 258 aa, 405 aa, and 527 aa positions (Fig. 4d). It has been reported that gene active sites are more easily to be docked by enzymes or ligands, which can regulate the genes’ expression. Therefore, this information is important on target sites for regulatory effects of gene expression based on the controlling of the target genes’ active sites.

## Discussion

At present, bovine mastitis is one of the most expensive diseases influencing animal wellbeing. The estimated monetary effect of mastitis in dairy cows on the US economy is 2 billion US dollars of annual lost income [22]. Various microorganisms can cause mastitis, and the sign of the illness can extend from totally subclinical to extreme and hazardous. For instance, *Escherichia coli* is one of the fundamental pathogens that are responsible for mastitis with its clinical signs in dairy cows. Approximately 5 to 20% of mammary-pathogenic *E*. *coli* (MPEC) infections are persistent [22].

Nonetheless, the cure rate of *E*. *coli* mastitis is low, and the pathogenesis of *E*. *coli* mastitis is not known. Even though the transcriptional reaction of cow mammary gland cells to in vitro infection has been investigated, the interaction and outcomes of these reactions in the in vivo condition of the mammary organ are less clear. Therefore, to generate new procedures and strategies for the rapid diagnosis of *E*. *coli* mastitis, an extensive molecular investigation of *E*. *coli* mastitis is required. This study coordinated microarray information to distinguish the potential key applicant genes in the dairy cow involved in the pathogenesis of *E*. *coli* mastitis [15].

MicroRNAs (miRNAs) assume a pivotal role in controlling inborn and versatile immunity in humans and animals [23]. An aggregate of 1838 miRNAs was recognized, including 580 known-miRNAs (incorporated for the miRbase database) and 1258 anticipated novel miRNAs. The miRNA articulation designs demonstrated that contrasted and control tests, 279 miRNAs and 305 miRNAs were differentially expressed miRNAs (DIE-miRNA) in *Staphylococcus aureus* and *E. coli-*infected tissues [24]. In which, Santos et al. [4] concluded that transcriptomics combined with bioinformatics analysis proved the suitability of the discovered drug treatment for recovery from the infectious bovine mastitis by interfering in the mechanisms of action of bovine mammary genes like MTOR and TP53.

In the present study, 4 DEGs in the dataset of GSE15441 were identified from 4 different bovine mammary regions that were infected with *E*. *coli*. These DEGs sets shared 101 ODEGs (100 upregulated and 1 downregulated gene), as listed in Table S1. According to the GO BPs and KEGG pathways enrichment analyses, the vast majority of BPs were immune-related, like GO:0006955~immune response, GO:0045087~innate immune response, and GO:0006954~ inflammatory response. Meanwhile, the DEG sets (23 top genes were involved) were significantly (0.05 > *P* > 0.001) related to the immune response, like IL10, IL8, and IL18. In which, the results confirmed that the immune response was closely related to bovine mastitis. Mandefro et al. [25] mentioned that the use of bovine immunological genes and their polymorphism could be the best disease markers in immunological traits of cattle. In which, a recent study showed that *E*. *coli* metabolites are involved in modulating immune functions of its host mammary glands [26]. That mastitis-associated *E*. *coli* harbors a higher number of genes for nutrient transport and carbohydrate metabolism through the TCA cycle [27]. The methyl-accepting chemotaxis proteins (MCP) or genes (tar, tsr, and tap) act through transmembrane receptors that mediate chemotactic response and cell-to-cell communication of *E*. *coli* [27]. The activation of the tumor repressor protein, p53, appears to play a central role in modulating the expression of various mammary epithelial cells involution responsive genes [28]. Thus, at the molecular level of the mammary gland immune response during infection by pathogenic bacteria, it is fundamental to design an effective therapy to control and eradicate bovine mastitis [29]. Therefore, the uniqueness of DEGs is that they are participating in the immune response-related BPs to genetically control the bovine mastitis problem. Among the 23 genes which participated in the immune response, IL8, IL18, IL1B, and PYDC1 were identified as being significant in the NOD-like receptor signaling pathway. These NOD-like receptors play an important role in host defense against the infection with *E*. *coli* mastitis [30]. Impairment of the NOD1/NF-κB pathway tends to downregulate mRNA levels of chemokine IL-8 [31]. As we further narrowed the scope of the important DEGs by finding the most important genes (CTSC, IL10, IL8, and IL18) (Fig. 3) [32]. In which, the integration between these DEGs and the reported bovine mastitis related miRNAs bta-miR-146a [33], bta-miR-21-5p, bta-miR-31 [5], bta-miR-16a, and bta-miR-155 [14] were done (Fig. 3). Furthermore, CTSC, IL10, IL8, and IL18 were verified to have the same expression pattern in GSE15441 and in GSE15202 [34]. In which, it suggests that their over-expression patterns in mammary tissue were not accidental but from the tendency of the representative biological targets.

## Conclusions

In this study, 2263 DEGs aggregates were identified in the different regions of the mammary gland (teat cistern, gland cistern, lobuloalveolar, and Furstenberg’s rosette). That resulted in 453, 597, 577, and 636 DEGs, respectively. Through bioinformatics investigation, IL8, IL10, IL18, and CTSC were recognized as the essential biomarkers for *E*. *coli* mastitis and were related to the immune response of bovine mastitis. This study provides potential key information for using the target genes as biomarkers of early cattle mastitis diagnosis. Also, further integrating of the DEGs and the TF that related to the immune response of the infecting pathogens was established to facilitate the development of the target drugs for controlling the transcription pathways of the infected animals against *E*. *coli* mastitis.

## Supplementary Information

**Additional file 1: Fig S1.** (a) Boxplot of GSE15441 data preprocessing before and after normalization. (b) Boxplot of GSE15020 data preprocessing before and after normalization. Blue and grey boxes refer to E. coli infected and healthy mammary tissue samples. **Fig S2.** Hierarchical clustering of DEGs in (a) teat cistern, (b) gland cistern, (c) lobuloalveolar, and (d) Furstenberg’s rosette. **Fig S3.** (a) The histogram of the category of enriched GO BP terms for the 101 ODEGs. (b) The histogram of the category of enriched KEGG pathways for the 101 Overlapping Differentially Express Genes (ODEGs). The horizontal axis represents the number of genes, and the vertical axis represents terms. The color bar means changes of significance. **Fig S4.** PPI network of Overlapping Differentially Express Genes (ODEGs). **Table S1.** Presents the 101 overlapped DEGs (ODEG; 100 up-regulated and 1 down-regulated the gene). **Table S2.** Enriched GO BPs for 101 ODEGs. **Table S3.** List of searched miRNAs from the miRTarBase database. **Table S4.** List of searched TFs from WebGestalt. **Table S5.** Copy Number Variants (CNVs) of CTSC, IL10, IL8, and IL18. **Table S6.** Single nucleotide polymorphisms (SNPs) of CTSC, IL10, IL8, and IL18.

## Data Availability

All the data and their availability are included in the manuscript text, figures, tables, and supplementary materials. Also, all relevant raw data will be freely available to any scientist wishing to use them for non-commercial purposes, without breaching.

## Abbreviations

DEG: Differentially expressed genes
ODEG: Overlapping differentially expressed genes
IL: Interleukin
IL1B: Interleukin 1 beta
CTSC: Cathepsin C
PYDC1: Pyrin domain-containing protein 1
PTK: Protein tyrosine kinase
NCF: Neutrophil cytosolic factor
CCR: C-C motif chemokine receptor
HCK: Hemopoetic cell kinase
miRNAs: Micro ribonucleic acid
TFs: Transcription factors
3D: Three dimension
*E*. *coli*: *Escherichia coli*
TLR: Toll-like receptor
TGFBI: Transforming growth factor beta induced
RT-PCR: Reverse transcriptase polymerase chain reaction
STH: Small-tailed sheep
GAM: Gansu Alpine Merino
GO: Gene ontogeny
KEGG: Kyoto Encyclopedia of Genes and Genomes
JAK-STAT: JAK (Janus tyrosine kinase)-STAT (signal transducer and activator of tranion)
MAPK: Mitogen-activated protein kinase
PI3K-Akt: Phosphatidylinositol-3-kinase
GEO: Gene expression omnibus
GSE: Genetics selection evolution
LIMMA: Linear models for microarray data
DAVID: Database for Annotation Visualization and Integrated Discovery
PPI: Protein-protein interaction
SMART: Simple Modular Architecture Research Tool
SNP: Single-nucleotide polymorphism
CNV: Copy number variants
