# Supplementary material for: Transcriptomic in silico analysis of bovine Escherichia coli mastitis highlights its immune-related expressed genes as an effective biomarker
Source: J Genet Eng Biotechnol. 2021 Oct 12;19:153. doi: 10.1186/s43141-021-00235-x (PMC8511192; doi:10.1186/s43141-021-00235-x)
Supplement: Supplementary file 1 — Additional file 1: Fig S1. (a) Boxplot of GSE15441 data preprocessing before and after normalization. (b) Boxplot of GSE15020 data preprocessing before and after normalization. Blue and grey boxes refer to E. coli infected and healthy mammary tissue samples. Fig S2. Hierarchical clustering of DEGs in (a) teat cistern, (b) gland cistern, (c) lobuloalveolar, and (d) Furstenberg’s rosette. Fig S3. (a) The histogram of the category of enriched GO BP terms for the 101 ODEGs. (b) The histogram of the category of enriched KEGG pathways for the 101 Overlapping Differentially Express Genes (ODEGs). The horizontal axis represents the number of genes, and the vertical axis represents terms. The color bar means changes of significance. Fig S4. PPI network of Overlapping Differentially Express Genes (ODEGs). Table S1. Presents the 101 overlapped DEGs (ODEG; 100 up-regulated and 1 down-regulated the gene). Table S2. Enriched GO BPs for 101 ODEGs. Table S3. List of searched miRNAs from the miRTarBase database. Table S4. List of searched TFs from WebGestalt. Table S5. Copy Number Variants (CNVs) of CTSC, IL10, IL8, and IL18. Table S6. Single nucleotide polymorphisms (SNPs) of CTSC, IL10, IL8, and IL18. [file 43141_2021_235_MOESM1_ESM.docx]

**
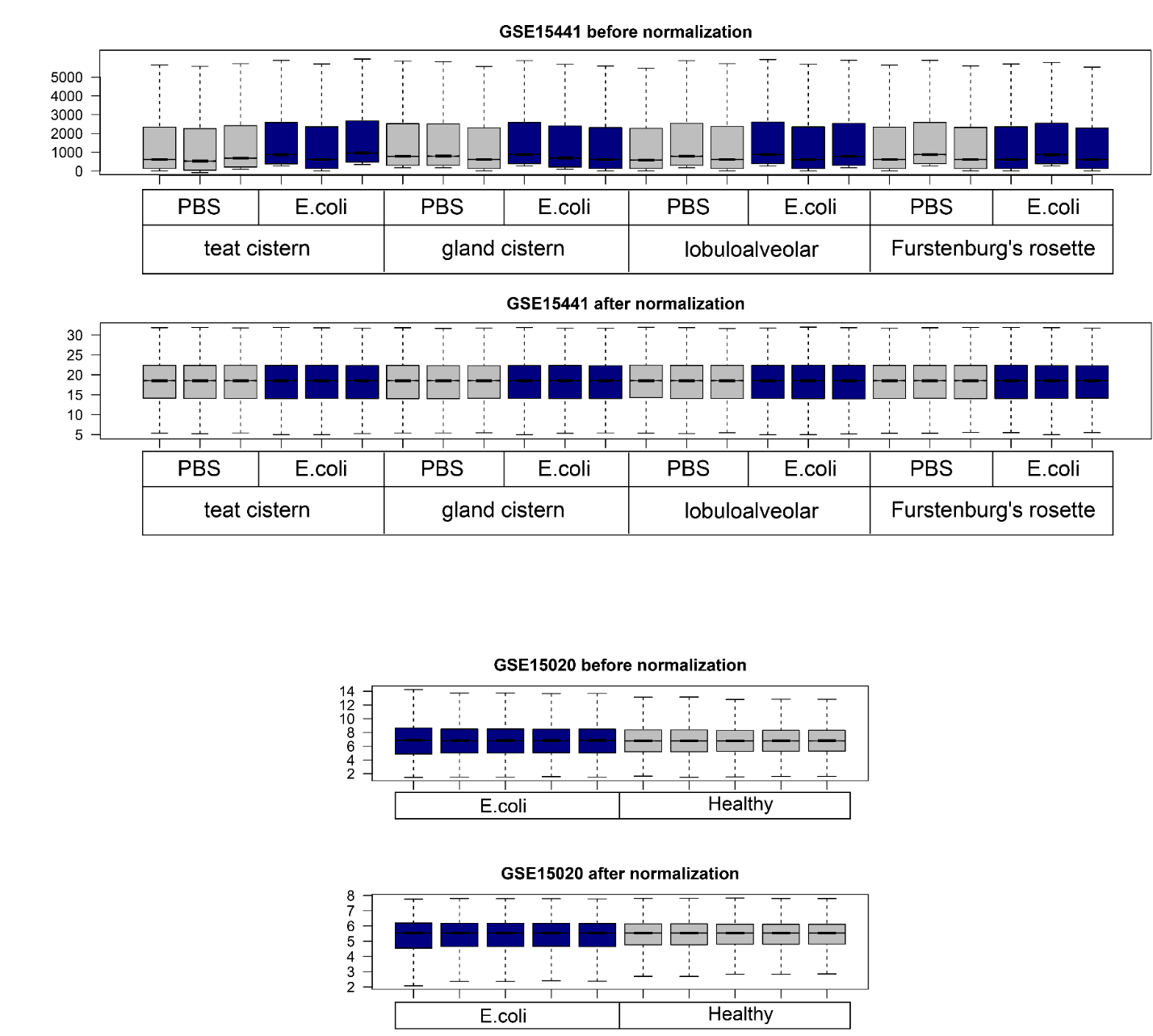
**

**(a)**

**(b)**

**Fig S1.** (**a**) Boxplot of GSE15441 data preprocessing before and after normalization. (**b**) Boxplot of GSE15020 data preprocessing before and after normalization. Blue and grey boxes refer to E. coli infected and healthy mammary tissue samples.

**(b)**

**(d)**

**(c)**

**(a)**


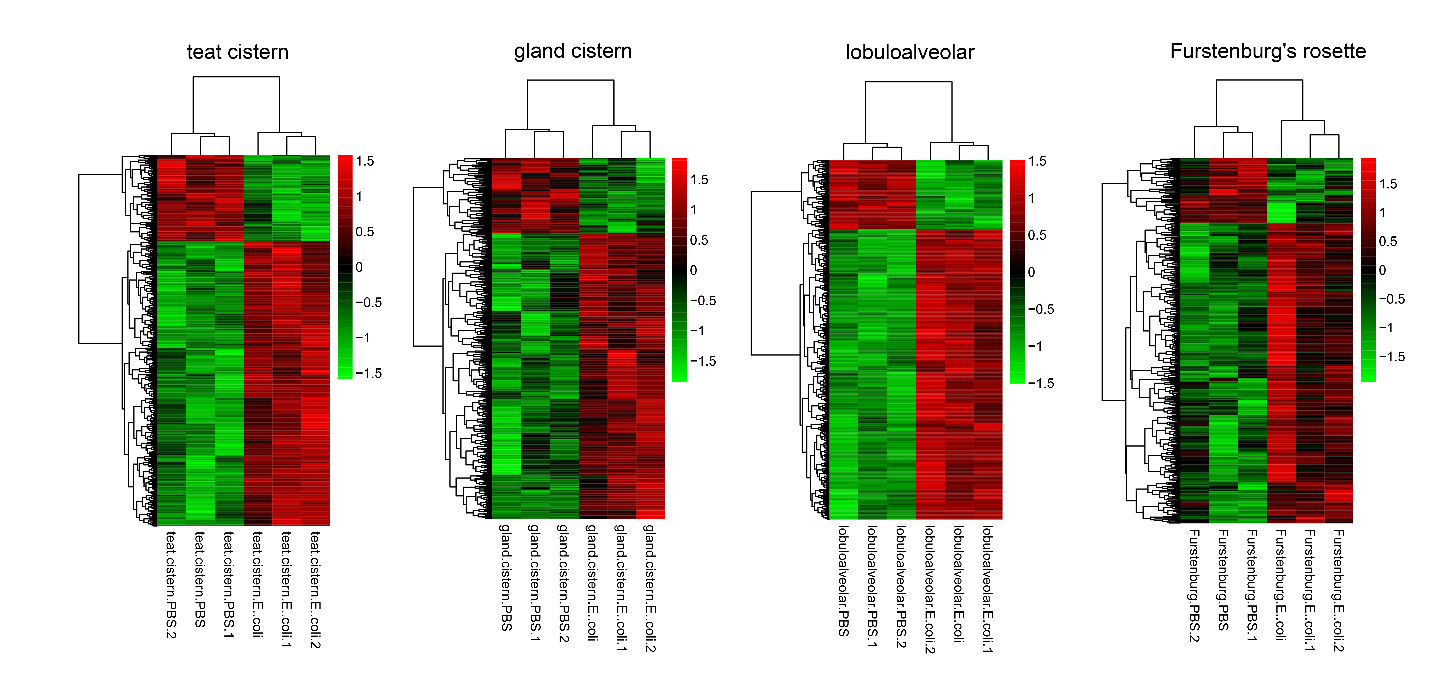


**Fig S2.** Hierarchical clustering of DEGs in (a) teat cistern, (b) gland cistern, (c) lobuloalveolar, and (d) Furstenberg’s rosette.

**(a)**


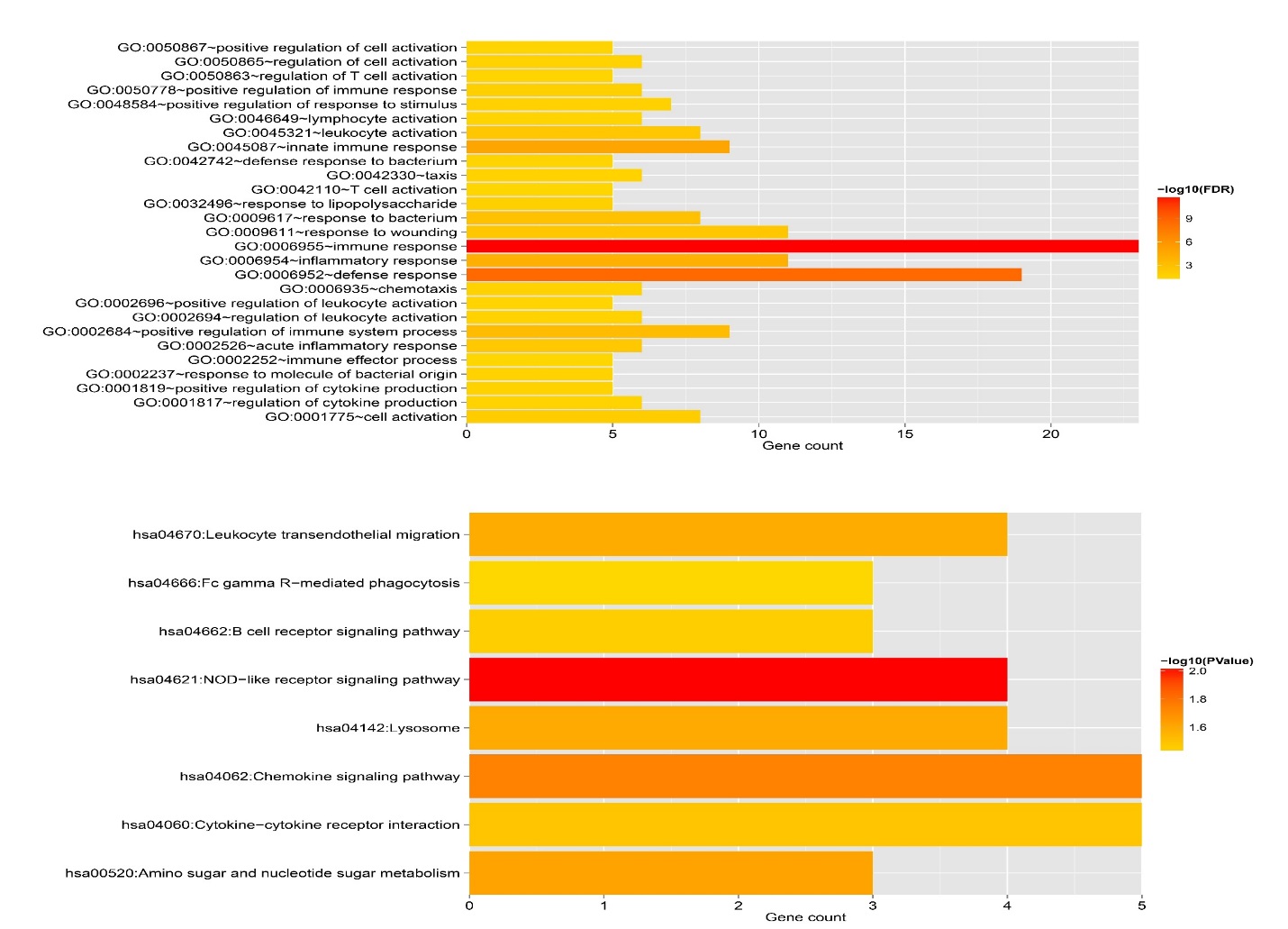


**(b)**

**Fig S3.** (**a)** The histogram of the category of enriched GO BP terms for the 101 ODEGs. (**b**) The histogram of the category of enriched KEGG pathways for the 101 Overlapping Differentially Express Genes (ODEGs). The horizontal axis represents the number of genes, and the vertical axis represents terms. The color bar means changes of significance.


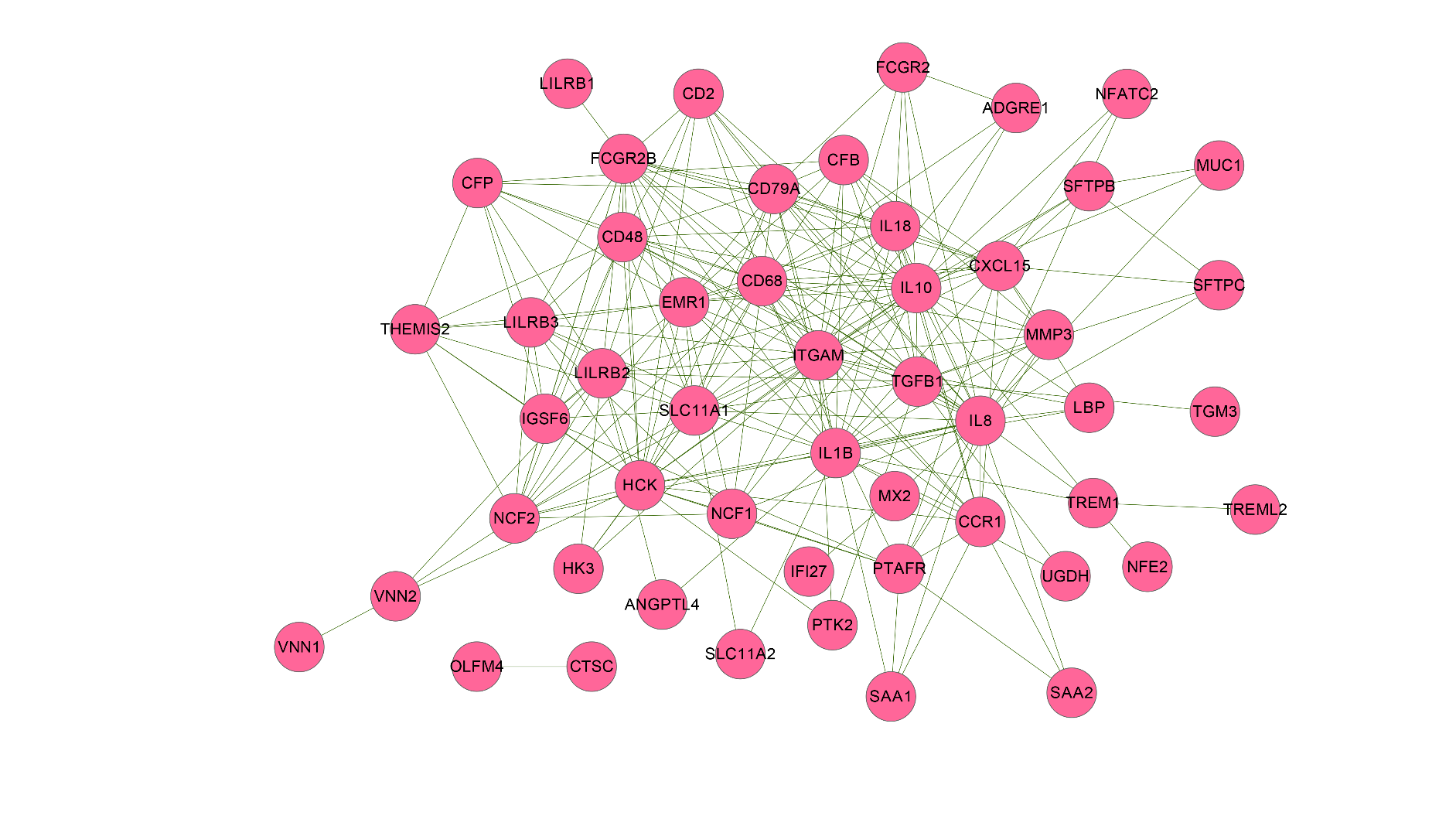


**Fig S4.** PPI network of Overlapping Differentially Express Genes (ODEGs).

| **Table S1. Presents the 101 overlapped DEGs (ODEG; 100 up-regulated and 1 down-regulated the gene).** | | | | | |
| --- | --- | --- | --- | --- | --- |
| **ID** | **FDR** | **p** | **logFC** | **Symbol** | **Characteristics** |
| **BOVINE0001S00000258** | 0.04975 | 0.0269 | 1.16319 | IL18 | TC267563 homologue to GB#AAR90937.1#40794748#AY513606 cytochrome P450 2A13 variant 3 {Homo sapiens;} , partial (53%) |
| **BOVINE0001S00000664** | 0.04975 | 0.0107 | 1.49061 | NL2 | TC286810 similar to UP#O43585 (O43585) CD2 binding protein 1 short form, partial (27%) |
| **BOVINE0001S00000797** | 0.04975 | 0.01109 | 1.30259 | IFI27 | TC295441 similar to UP#PU1_HUMAN (P17947) 31 kDa transforming protein (Transcription factor PU.1), partial (59%) |
| **BOVINE0001S00001167** | 0.04975 | 0.0395 | 1.31218 | LILRB2 | TC260197 UP#Q864S1 (Q864S1) Cathepsin C (Fragment), complete |
| **BOVINE0001S00001229** | 0.04975 | 0.00766 | 1.0624 | CTSC | TC271409 similar to UP#Q969Q4 (Q969Q4) ADP-ribosylation factor-like 11, partial (46%) |
| **BOVINE0001S00001299** | 0.04975 | 0.00495 | 1.33582 | SCO2 | TC293828 weakly similar to GB#AAC51901.1#2665643#AF031554 immunoglobulin-like transcript 5 {Homo sapiens;} , partial (37%) |
| **BOVINE0001S00001563** | 0.04975 | 0.00346 | 1.8371 | IgA | TC275396 similar to UP#Q8CFT8 (Q8CFT8) Chchd2 protein (Fragment), partial (10%) |
| **BOVINE0001S00002506** | 0.03925 | 0.00104 | 1.77535 | MSDA | TC291738 homologue to GB#CAB51288.1#5578776#HSA243937 G18.1b protein {Homo sapiens;} , complete |
| **BOVINE0001S00002557** | 0.04595 | 0.00204 | 1.61071 | ACADVL | TC290861 UP#UGDH_BOVIN (P12378) UDP-glucose 6-dehydrogenase (UDP-Glc dehydrogenase) (UDP-GlcDH) (UDPGDH) , complete |
| **BOVINE0001S00002674** | 0.04975 | 0.0038 | 1.24529 | CFB | TC295250 similar to GB#CAA10569.1#4128049#HSA132100 VNN2 protein {Homo sapiens;} , partial (21%) |
| **BOVINE0001S00003313** | 0.04915 | 0.00281 | 1.27702 | CD68 | TC270549 UP#Q9TTY5 (Q9TTY5) Platelet-activating factor receptor, complete |
| **BOVINE0001S00003890** | 0.04975 | 0.02801 | 1.53092 | LEM1 | TC282999 similar to UP#VNN2_HUMAN (O95498) Vascular non-inflammatory molecule 2 precursor (Vanin 2) (Glycosylphosphatidyl inositol-anchored protein GPI-80) (FOAP-4 protein), partial (77%) |
| **BOVINE0001S00004805** | 0.04745 | 0.00243 | 1.60432 | FCGR2B | TC265721 weakly similar to GB#AAA62834.1#180139#HUMCD48 pan-leukocyte antigen {Homo sapiens;} , partial (62%) |
| **BOVINE0001S00004866** | 0.0267 | 0.00029 | 3.63481 | LAP | TC260507 similar to GB#AAD17190.1#4324928#AF110799 interleukin-18 binding protein a precursor {Homo sapiens;} , partial (73%) |
| **BOVINE0001S00005073** | 0.04975 | 0.02977 | 1.09222 | CHCHD2 | TC287953 weakly similar to UP#SIL5_HUMAN (O15389) Sialic acid binding Ig-like lectin 5 precursor (Siglec-5) (Obesity-binding protein 2) (OB binding protein-2) (OB-BP2) (CD33 antigen-like 2) (CD170 antigen), partial (17%) |
| **BOVINE0001S00005299** | 0.04975 | 0.00626 | 2.2517 | PTTG1IP | TC262854 similar to UP#CFAB_PANTR (Q864W0) Complement factor B precursor (C3/C5 convertase) , partial (25%) |
| **BOVINE0001S00005300** | 0.04975 | 0.0043 | 2.08225 | AD08 | TC273588 similar to UP#Q7Q2P0 (Q7Q2P0) AgCP2776 (Fragment), partial (5%) |
| **BOVINE0001S00005301** | 0.03805 | 0.00078 | 1.73945 | AD08 | TC288396 similar to UP#Q6PK94 (Q6PK94) C13orf18 protein (Fragment), partial (11%) |
| **BOVINE0001S00005302** | 0.04975 | 0.00403 | 1.84699 | AD08 | TC263493 similar to UP#Q6RG76 (Q6RG76) CD68 (Fragment), partial (24%) |
| **BOVINE0001S00005335** | 0.04975 | 0.01368 | 1.42465 | KPNA4 | TC269965 similar to UP#Q91YX0 (Q91YX0) BC013712 protein (Fragment), partial (13%) |
| **BOVINE0001S00005853** | 0.04975 | 0.00371 | 2.24165 | PTK2 | TC268762 similar to GB#BAD12835.1#45597289#AB120412 leukocyte immunoglobulin-like receptor {Equus caballus;} , partial (24%) |
| **BOVINE0001S00005914** | 0.04975 | 0.01206 | 1.49498 | NCF2 | TC261046 homologue to UP#Q95MQ2 (Q95MQ2) Inteferon-induced membrane protein Leu-13/9-27, partial (91%) |
| **BOVINE0001S00006020** | 0.03045 | 0.0005 | 3.29867 | GCSR | TC261416 similar to UP#Q8MJZ3 (Q8MJZ3) Leukocyte immunoglobulin-like receptor d, partial (8%) |
| **BOVINE0001S00006021** | 0.02625 | 0.00024 | 2.21487 | GCSR | TC274676 similar to GB#AAB63280.1#2257939#AF005665 properdin {Homo sapiens;} , partial (27%) |
| **BOVINE0001S00006047** | 0.04975 | 0.02014 | 1.1445 | CD48 | TC282520 weakly similar to GB#AAH57951.1#37046824#BC057951 immunoglobulin superfamily, member 6 {Mus musculus;} , partial (59%) |
| **BOVINE0001S00006387** | 0.04615 | 0.00225 | 2.63135 | SLC11A1 | TC261479 homologue to PDB#1V54_B.0#40889824#1V54_B Chain B, Bovine Heart Cytochrome C Oxidase At The Fully Oxidized State. {Bos taurus;} , partial (50%) |
| **BOVINE0001S00006539** | 0.04975 | 0.00774 | 2.08196 | MM01 | TC272507 homologue to UP#Q8BY15 (Q8BY15) Mus musculus 3 days neonate thymus cDNA, RIKEN full-length enriched library, clone:A630097K09 product:SORTING NEXIN 10, full insert sequence, partial (11%) |
| **BOVINE0001S00006572** | 0.03955 | 0.00117 | 1.64395 | CBS138 | TC265009 homologue to UP#Q96KW7 (Q96KW7) KPNA4 protein, partial (16%) |
| **BOVINE0001S00006862** | 0.03925 | 0.00098 | 1.60198 | PABPN1L | TC286505 UP#LEM1_BOVIN (P98131) L-selectin precursor (Lymph node homing receptor) (Leukocyte adhesion molecule-1) (LAM-1) (Leukocyte-endothelial cell adhesion molecule 1) (LECAM1) (CD62L), partial (41%) |
| **BOVINE0001S00006958** | 0.04975 | 0.00666 | 1.78994 | SFTPB | TC276510 similar to UP#PGSG_HUMAN (P10124) Secretory granule proteoglycan core protein precursor (Platelet proteoglycan core protein) (P.PG) (Hematopoetic proteoglycan core protein) (Serglycin), partial (21%) |
| **BOVINE0001S00007721** | 0.04975 | 0.00504 | 1.50741 | LILRB1 | TC297443 UP#Q93U40 (Q93U40) RNA polymerase beta subunit (Fragment), partial (20%) |
| **BOVINE0001S00007789** | 0.03805 | 0.00085 | 2.67501 | TREML2 | TC293954 similar to GB#AAQ89176.1#37182752#AY358817 EMR3 {Homo sapiens;} , partial (31%) |
| **BOVINE0001S00007889** | 0.03045 | 0.0005 | -5.1802 | CYP2A13 | TC283890 similar to UP#TGM3_HUMAN (Q08188) Protein-glutamine glutamyltransferase E precursor (TGase E) (TGE) (TG(E)) (Transglutaminase 3) , partial (31%) |
| **BOVINE0001S00008001** | 0.02625 | 0.00026 | 3.45676 | ADGRE1 | TC260913 similar to UP#Q6UXN0 (Q6UXN0) NL2, partial (93%) |
| **BOVINE0001S00008290** | 0.02625 | 0.00025 | 3.03375 | TREM-1 | TC265588 UP#Q95L72 (Q95L72) NADPH oxidase cytosolic protein p40phox, partial (48%) |
| **BOVINE0001S00009014** | 0.04975 | 0.00435 | 1.30077 | LILRB3 | TC280407 homologue to UP#IL10_BOVIN (P43480) Interleukin-10 precursor (IL-10) (Cytokine synthesis inhibitory factor) (CSIF), complete |
| **BOVINE0001S00009129** | 0.0434 | 0.00153 | 2.02591 | HCK | TC267395 weakly similar to UP#Q8MJZ3 (Q8MJZ3) Leukocyte immunoglobulin-like receptor d, partial (15%) |
| **BOVINE0001S00009684** | 0.02625 | 0.00012 | 2.0442 | SFTPC | TC264070 UP#LEM1_BOVIN (P98131) L-selectin precursor (Lymph node homing receptor) (Leukocyte adhesion molecule-1) (LAM-1) (Leukocyte-endothelial cell adhesion molecule 1) (LECAM1) (CD62L), partial (98%) |
| **BOVINE0001S00010217** | 0.04975 | 0.00662 | 1.27712 | BC013712 | TC275335 weakly similar to UP#NFAM_HUMAN (Q8NET5) NFAT activation molecule 1 precursor (Calcineurin/NFAT-activating ITAM-containing protein), partial (79%) |
| **BOVINE0001S00010801** | 0.04975 | 0.00304 | 1.14232 | PTAFR | TC293128 homologue to GB#AAC50422.1#1147781#HSU42303 ATP:D-hexose 6-phosphotransferase {Homo sapiens;} , partial (53%) |
| **BOVINE0001S00011587** | 0.04975 | 0.01373 | 1.07725 | ARL11 | TC266536 similar to UP#Q98SP8 (Q98SP8) Embryonic poly(A) binding protein, partial (44%) |
| **BOVINE0001S00012685** | 0.04975 | 0.00334 | 1.3443 | SNX10 | TC268632 UP#Q28109 (Q28109) FC gamma 2 receptor, complete |
| **BOVINE0001S00013766** | 0.04975 | 0.04318 | 1.24949 | AgCP2776 | TC262737 homologue to GB#AAD47199.1#5702202#AF129166 long-chain acyl-CoA synthetase 5 {Homo sapiens;} , partial (57%) |
| **BOVINE0001S00014454** | 0.04345 | 0.00166 | 2.37299 | SY19 | TC292144 homologue to UP#CYH4_HUMAN (Q9UIA0) Cytohesin 4, partial (18%) |
| **BOVINE0001S00014507** | 0.03045 | 0.00044 | 3.72289 | FCN3 | TC266246 homologue to UP#Q6FL51 (Q6FL51) Strain CBS138 chromosome L complete sequence, partial (10%) |
| **BOVINE0001S00014778** | 0.04975 | 0.04091 | 1.31776 | CFP | TC286809 homologue to UP#CYH4_HUMAN (Q9UIA0) Cytohesin 4, partial (55%) |
| **BOVINE0001S00015220** | 0.03925 | 0.00108 | 1.86253 |  | TC296373 similar to UP#Q7Z6F8 (Q7Z6F8) MGC18079, partial (7%) |
| **BOVINE0001S00015366** | 0.04975 | 0.03314 | 1.57319 | NFATC2 | TC291893 UP#NCF1_BOVIN (O77774) Neutrophil cytosol factor 1 (NCF-1) (Neutrophil NADPH oxidase factor 1) (47 kDa neutrophil oxidase factor) (p47-phox) (NCF-47K), complete |
| **BOVINE0001S00015558** | 0.03045 | 0.00048 | 1.74494 |  | TC299834 UP#NRM1_BISBI (Q95102) Natural resistance-associated macrophage protein 1 (NRAMP 1), partial (23%) |
| **BOVINE0001S00016062** | 0.0149 | 1.3E-05 | 3.47788 | C3L2 | TC294791 chemokine C-C motif receptor 1 [Bos taurus] |
| **BOVINE0001S00016132** | 0.03955 | 0.00115 | 1.97535 | CHIT1 | TC264975 similar to UP#AD08_HUMAN (P78325) ADAM 8 precursor (A disintegrin and metalloproteinase domain 8) (Cell surface antigen MS2) (CD156a antigen) (CD156) , partial (13%) |
| **BOVINE0001S00016399** | 0.0434 | 0.00159 | 1.44748 | PGSG | TC275598 weakly similar to UP#O97916 (O97916) Reverse transcriptase-like, partial (7%) |
| **BOVINE0001S00017717** | 0.04975 | 0.00606 | 2.15471 | IL1B | TC292784 UP#NCF2_BOVIN (O77775) Neutrophil cytosol factor 2 (NCF-2) (Neutrophil NADPH oxidase factor 2) (67 kDa neutrophil oxidase factor) (p67-phox), complete |
| **BOVINE0001S00019176** | 0.04975 | 0.00906 | 2.56481 | VNN1 | TC262686 GB#AAA30655.1#498260#BOVMSDA manganous superoxide dismutase {Bos taurus;} , partial (80%) |
| **BOVINE0001S00019371** | 0.04975 | 0.0101 | 1.82275 | TGM3 | TC266632 similar to UP#Q9XSA0 (Q9XSA0) Pulmonary surfactant-associated protein B (Fragment), partial (12%) |
| **BOVINE0001S00019568** | 0.04975 | 0.00725 | 4.13711 | IL8 | TC295381 similar to GB#AAB93671.1#2734092#AC003965 SP001LA {Homo sapiens;} , partial (65%) |
| **BOVINE0001S00019797** | 0.04675 | 0.00238 | 1.97701 | NFE2 | TC285086 UP#BD05_BOVIN (P46163) Beta-defensin 5 precursor (BNDB-5) (BNBD-5), complete |
| **BOVINE0001S00019966** | 0.03925 | 0.00103 | 2.50969 | OLFM4 | TC279100 similar to PDB#1L9N_A.0#21466001#1L9N_A Chain A, Three-Dimensional Structure Of The Human Transglutaminase 3 Enzyme: Binding Of Calcium Ions Change Structure For Activation. {Homo sapiens;} , partial (36%) |
| **BOVINE0001S00020678** | 0.03925 | 0.00105 | 1.49587 | IL10 | TC261743 GB#AAP41842.1#32396010#AY247821 immunoglobulin A Fc receptor {Bos taurus;} , complete |
| **BOVINE0001S00021603** | 0.02625 | 6.7E-05 | 1.98291 | BD04 | TC291625 similar to UP#MM19_HUMAN (Q99542) Matrix metalloproteinase-19 precursor (MMP-19) (Matrix metalloproteinase RASI) (MMP-18) , partial (92%) |
| **BOVINE0001S00022591** | 0.03805 | 0.00086 | 1.98259 | EMR3 | TC264976 weakly similar to UP#AD08_MOUSE (Q05910) ADAM 8 precursor (A disintegrin and metalloproteinase domain 8) (Cell surface antigen MS2) (Macrophage cysteine-rich glycoprotein) (CD156 antigen) , partial (10%) |
| **BOVINE0001S00022791** | 0.04975 | 0.00351 | 1.33114 | IGSF6 | TC297013 homologue to UP#Q95N32 (Q95N32) Pyrin (Fragment), partial (9%) |
| **BOVINE0001S00023270** | 0.04975 | 0.03177 | 1.14342 | VNN2 | TC275189 weakly similar to UP#Q7S078 (Q7S078) Predicted protein, partial (4%) |
| **BOVINE0001S00023777** | 0.04455 | 0.00194 | 1.98937 | ITGAM | TC297341 similar to UP#TR6B_HUMAN (O95407) Tumor necrosis factor receptor superfamily member 6B precursor (Decoy receptor for Fas ligand) (Decoy receptor 3) (DcR3) (M68) (UNQ186/PRO212), partial (55%) |
| **BOVINE0001S00024161** | 0.04975 | 0.01293 | 1.4856 | TGM3 | TC293836 similar to GB#AAB40595.1#1754987#ATU43946 strictosidine synthase {Arabidopsis thaliana;} , partial (5%) |
| **BOVINE0001S00025282** | 0.02625 | 0.00015 | 1.81602 | BD05 | TC288677 similar to UP#LBP_HUMAN (P18428) Lipopolysaccharide-binding protein precursor (LBP), partial (89%) |
| **BOVINE0001S00026622** | 0.04975 | 0.01639 | 1.43629 | LEM1 | TC276243 similar to GB#AAB04534.1#1439568#HSU58515 chitinase {Homo sapiens;} , partial (19%) |
| **BOVINE0001S00026804** | 0.03045 | 0.00053 | 2.67796 | MX2 | TC279526 homologue to UP#NFE2_HUMAN (Q16621) Transcription factor NF-E2 45 kDa subunit (Nuclear factor, erythroid-derived 2 45 kDa subunit) (P45 NF-E2) (Leucine zipper protein NF-E2), complete |
| **BOVINE0001S00026926** | 0.02625 | 0.0001 | 1.67646 | CYH4 | TC282320 weakly similar to GB#AAQ89176.1#37182752#AY358817 EMR3 {Homo sapiens;} , partial (26%) |
| **BOVINE0001S00026927** | 0.04975 | 0.04551 | 1.02942 | CD2 | TC281332 UP#BD04_BOVIN (P46162) Beta-defensin 4 precursor (BNDB-4) (BNBD-4), complete |
| **BOVINE0001S00027993** | 0.04975 | 0.00309 | 1.16396 | SIL5 | TC283506 similar to GB#AAA59903.1#386975#HUMNARIA neutrophil adherence receptor alpha-M subunit {Homo sapiens;} , partial (18%) |
| **BOVINE0001S00028219** | 0.04975 | 0.00462 | 2.52196 | PIR-A2 | TC268877 similar to UP#HCK_HUMAN (P08631) Tyrosine-protein kinase HCK (p59-HCK/p60-HCK) (Hemopoietic cell kinase) , partial (22%) |
| **BOVINE0001S00028436** | 0.04975 | 0.00597 | 1.27451 | C13orf18 | TC269432 similar to UP#Q9XSA0 (Q9XSA0) Pulmonary surfactant-associated protein B (Fragment), partial (14%) |
| **BOVINE0001S00028717** | 0.04455 | 0.00186 | 1.97483 | LBP | TC294023 similar to GB#AAH20614.1#18089045#BC020614 inflammation-related G protein-coupled receptor EX33 {Homo sapiens;} , complete |
| **BOVINE0001S00028839** | 0.03015 | 0.00036 | 2.99737 | SLC7A3 | TC266213 UP#MM01_BOVIN (P28053) Interstitial collagenase precursor (Matrix metalloproteinase-1) (MMP-1) (Fibroblast collagenase) , complete |
| **BOVINE0001S00029798** | 0.04615 | 0.00231 | 2.22543 | MUC1 | TC264974 similar to UP#AD08_HUMAN (P78325) ADAM 8 precursor (A disintegrin and metalloproteinase domain 8) (Cell surface antigen MS2) (CD156a antigen) (CD156) , partial (7%) |
| **BOVINE0001S00030237** | 0.04975 | 0.00442 | 2.30644 | SAA | TC278459 GB#AAA30584.1#163201#BOVIL1B interleukin 1-beta {Bos taurus;} , complete |
| **BOVINE0001S00030901** | 0.04975 | 0.02518 | 1.11807 | UGDH | TC265695 similar to UP#GCSR_HUMAN (Q99062) Granulocyte colony stimulating factor receptor precursor (G-CSF-R) (CD114 antigen), partial (60%) |
| **BOVINE0001S00031421** | 0.02625 | 0.00017 | 4.20584 | SDHL | TC289758 mucin |
| **BOVINE0001S00031665** | 0.02625 | 0.00026 | 1.84609 | MM19 | TC265527 homologue to GB#AAA52643.1#306832#HUMHCKA protein-tyrosine kinase {Homo sapiens;} , partial (65%) |
| **BOVINE0001S00031728** | 0.04975 | 0.00542 | 3.14279 | SZ06 | TC264973 similar to GB#AAF73770.1#8163626#AF149785 pituitary tumor-transforming gene protein binding factor {Homo sapiens;} , partial (7%) |
| **BOVINE0001S00031778** | 0.04975 | 0.01939 | 1.10192 | GPSM3 | TC290197 UP#SAA_BOVIN (P35541) Serum amyloid A protein (SAA) [Contains: Amyloid protein A (Amyloid fibril protein AA)], partial (49%) |
| **BOVINE0001S00031862** | 0.04975 | 0.00897 | 1.72131 | NCF1 | TC298717 weakly similar to UP#ICA2_HUMAN (P13598) Intercellular adhesion molecule-2 precursor (ICAM-2) (CD102 antigen), partial (14%) |
| **BOVINE0001S00032113** | 0.04975 | 0.00348 | 1.63907 | CYH4 | TC274352 similar to UP#SY19_MOUSE (O70460) Small inducible cytokine A19 precursor (CCL19) (Epstein-Barr virus induced molecule 1 ligand chemokine) (EBI1-ligand chemokine) (ELC), partial (69%) |
| **BOVINE0001S00032681** | 0.04975 | 0.00615 | 1.75283 | NCF2 | TC294158 homologue to GB#AAD08636.1#4097862#OAU70255 natural resistance associated macrophage protein {Ovis aries;} , partial (26%) |
| **BOVINE0001S00033025** | 0.04975 | 0.02116 | 1.59993 | HK3 | TC279695 similar to UP#Q86T22 (Q86T22) GW112 protein (Fragment), complete |
| **BOVINE0001S00033725** | 0.04615 | 0.00232 | 1.09198 | ILT5 | TC288179 weakly similar to GB#AAD50906.1#5758945#AF169638 paired Ig-like receptor-A2 {Rattus norvegicus;} , partial (11%) |
| **BOVINE0001S00033733** | 0.0401 | 0.00123 | 1.92581 | MMP3 | TC278905 similar to UP#VNN1_PIG (Q9BDJ5) Pantetheinase precursor (Pantetheine hydrolase) (Vascular non-inflammatory molecule 1) (Vanin 1) , partial (94%) |
| **BOVINE0001S00033851** | 0.03955 | 0.00111 | 1.48207 | EMR3 | TC266061 homologue to GB#AAD08636.1#4097862#OAU70255 natural resistance associated macrophage protein {Ovis aries;} , complete |
| **BOVINE0001S00033920** | 0.04975 | 0.00319 | 2.06329 | GPR84 | TC267463 similar to UP#Q8IWY0 (Q8IWY0) TREM-like transcript 2 (APAF6268), partial (46%) |
| **BOVINE0001S00034055** | 0.03615 | 0.0007 | 2.46976 | SLC11A2 | TC286687 UP#Q9BDI7 (Q9BDI7) GTP-binding protein MX2, complete |
| **BOVINE0001S00034688** | 0.0481 | 0.00257 | 1.73534 | CCR1 | TC288799 similar to GB#AAH33816.1#21707300#BC033816 solute carrier family 7 (cationic amino acid transporter, y+ system), member 3 {Homo sapiens;} , partial (27%) |
| **BOVINE0001S00035147** | 0.04975 | 0.01663 | 1.1261 | VNN2 | TC267964 UP#Q6QUN5 (Q6QUN5) TREM-1, complete |
| **BOVINE0001S00035278** | 0.04975 | 0.00516 | 1.79195 | SP001LA | TC291688 UP#SZ06_BOVIN (P80221) Small inducible cytokine B6 precursor (CXCL6) (Granulocyte chemotactic protein 2) (GCP-2), complete |
| **BOVINE0001S00035338** | 0.04975 | 0.01388 | 1.04885 | PU1 | TC265694 similar to UP#GCSR_HUMAN (Q99062) Granulocyte colony stimulating factor receptor precursor (G-CSF-R) (CD114 antigen), partial (19%) |
| **BOVINE0001S00036270** | 0.04455 | 0.00187 | 1.70554 | MGC18079 | TC267675 similar to GB#AAH59395.1#37747859#BC059395 EMR1 protein {Homo sapiens;} , partial (4%) |
| **BOVINE0001S00036910** | 0.0496 | 0.00289 | 1.84731 | PYDC1 | TC276173 similar to UP#C3L2_HUMAN (Q15782) Chitinase 3-like protein 2 precursor (YKL-39) (Chondrocyte protein 39), complete |
| **BOVINE0001S00037238** | 0.04975 | 0.04157 | 1.90261 | TR6B | TC270660 UP#LAP_BOVIN (Q28880) Lingual antimicrobial peptide precursor, complete |
| **BOVINE0001S00037340** | 0.03955 | 0.00115 | 1.46011 |  | TC274405 similar to UP#Q29042 (Q29042) Ficolin, complete |
| **BOVINE0001S00038614** | 0.04975 | 0.00326 | 2.3585 | ICA2 | TC279297 UP#IL8_BOVIN (P79255) Interleukin-8 precursor (IL-8) (CXCL8), complete |
| **BOVINE0001S00039654** | 0.04975 | 0.01225 | 1.72405 | NRM1 | TC291381 similar to UP#SDHL_HUMAN (P20132) L-serine dehydratase (L-serine deaminase) , partial (98%) |

| Table S2. Enriched GO BPs for 101 ODEGs | | | |
| --- | --- | --- | --- |
| **Term** | **Count** | **FDR** | ***P*-Value** |
| **GO:0006955~immune response** | 23 | 1.47E-12 | 1.69E-15 |
| **GO:0006952~defense response** | 19 | 2.38E-09 | 5.40E-12 |
| **GO:0045087~innate immune response** | 9 | 1.45E-05 | 4.93E-08 |
| **GO:0006954~inflammatory response** | 11 | 7.93E-05 | 3.60E-07 |
| **GO:0002684~positive regulation of immune system process** | 9 | 5.54E-04 | 3.15E-06 |
| **GO:0009617~response to bacterium** | 8 | 0.00123 | 8.39E-06 |
| **GO:0009611~response to wounding** | 11 | 0.00352 | 2.81E-05 |
| **GO:0045321~leukocyte activation** | 8 | 0.00353 | 3.62E-05 |
| **GO:0002526~acute inflammatory response** | 6 | 0.00392 | 3.57E-05 |
| **GO:0001775~cell activation** | 8 | 0.0093 | 1.06E-04 |
| **GO:0050778~positive regulation of immune response** | 6 | 0.01664 | 2.29E-04 |
| **GO:0032496~response to lipopolysaccharide** | 5 | 0.01694 | 2.14E-04 |
| **GO:0048584~positive regulation of response to stimulus** | 7 | 0.01846 | 2.75E-04 |
| **GO:0001819~positive regulation of cytokine production** | 5 | 0.01992 | 3.89E-04 |
| **GO:0002237~response to molecule of bacterial origin** | 5 | 0.02032 | 3.27E-04 |
| **GO:0002694~regulation of leukocyte activation** | 6 | 0.02067 | 4.27E-04 |
| **GO:0006935~chemotaxis** | 6 | 0.02095 | 3.61E-04 |
| **GO:0042330~taxis** | 6 | 0.02095 | 3.61E-04 |
| **GO:0050865~regulation of cell activation** | 6 | 0.02365 | 5.44E-04 |
| **GO:0001817~regulation of cytokine production** | 6 | 0.02622 | 6.34E-04 |
| **GO:0002696~positive regulation of leukocyte activation** | 5 | 0.02848 | 7.22E-04 |
| **GO:0042742~defense response to bacterium** | 5 | 0.03077 | 8.88E-04 |
| **GO:0050867~positive regulation of cell activation** | 5 | 0.03099 | 8.58E-04 |
| **GO:0046649~lymphocyte activation** | 6 | 0.03236 | 9.71E-04 |
| **GO:0050863~regulation of T cell activation** | 5 | 0.03349 | 0.001045 |
| **GO:0042110~T cell activation** | 5 | 0.03956 | 0.001375 |
| **GO:0002252~immune effector process** | 5 | 0.04636 | 0.001725 |

| Table S3. List of searched miRNAs from the miRTarBase database. | |
| --- | --- |
| **DE target** | **miRNA** |
| **CCR1** | bta-miR-10a |
| **CFB** | bta-mir-26a,bta-let-7a,bta-mir-214 |
| **CTSC** | bta-miR-146a,bta-miR-21-5p,bta-miR-31,bta-mir-23b |
| **IL10** | bta-miR-146a,bta-miR-15b,bta-miR-16a |
| **IL18** | bta-miR-155,bta-miR-15b,bta-miR-16a,bta-miR-181a |
| **IL8** | bta-miR-146a,bta-miR-15b,bta-miR-16a,bta-miR-17-5p |
| **LBP** | bta-mir-142,bta-mir-145 |

| Table S4. List of searched TFs from WebGestalt. | | | | |
| --- | --- | --- | --- | --- |
| **TF** | **Gene count** | ***P*-value** | **FDR** | **Target DEGs** |
| **ETS2** | 9 | 2.99E-06 | 3.59E-05 | CD79A,FCGR2B,CCR1,MMP3,TREML2,NFATC2,IL10,NCF2,TGM3 |
| **AP1** | 8 | 3.20E-05 | 0.0002 | CD68,SLC11A1,MMP3,SFTPC,IL10,HK3,ANGPTL4,TGM3 |
| **ETS** | 4 | 0.0002 | 0.0005 | CD79A,MMP3,TREML2,PTK2 |
| **IRF** | 4 | 0.0002 | 0.0005 | FCGR2B,NCF1,SLC11A2,PTK2 |
| **HNF3** | 5 | 0.0014 | 0.0028 | CD68,SFTPC,SFTPB,IL18,CFB |
| **LEF1** | 6 | 0.0024 | 0.0041 | NFE2,CCR1,CTSC,ITGAM,VNN1,CFB |

| Table S5. Copy Number Variants (CNVs) of CTSC, IL10, IL8, and IL18. | | | |
| --- | --- | --- | --- |
| **Variant ID** | **Type** | **Subtype** | **PubMed ID** |
| **CNV for CTSC** | | | |
| nsv478403 | CNV | Novel sequence insertion | 20440878 |
| nsv1145559 | CNV | Deletion | 24896259 |
| esv3892054 | CNV | Loss | 25118596 |
| **CNV for IL8** |  |  |  |
| [nsv1001725](http://dgv.tcag.ca/dgv/app/variant?id=nsv1001725) | CNV | Gain | [25217958](http://www.ncbi.nlm.nih.gov/entrez/query.fcgi?db=PubMed&cmd=search&Dopt=b&term=25217958) |
| **CNV for IL18** |  |  |  |
| [nsv519192](http://dgv.tcag.ca/dgv/app/variant?id=nsv519192) | CNV | Gain | [19592680](http://www.ncbi.nlm.nih.gov/entrez/query.fcgi?db=PubMed&cmd=search&Dopt=b&term=19592680) |
| [nsv1134175](http://dgv.tcag.ca/dgv/app/variant?id=nsv1134175) | CNV | Deletion | [24896259](http://www.ncbi.nlm.nih.gov/entrez/query.fcgi?db=PubMed&cmd=search&Dopt=b&term=24896259) |
| [esv2745077](http://dgv.tcag.ca/dgv/app/variant?id=esv2745077) | CNV | Deletion | [23290073](http://www.ncbi.nlm.nih.gov/entrez/query.fcgi?db=PubMed&cmd=search&Dopt=b&term=23290073) |
| **CNV for IL10** |  |  |  |
| [esv3578391](http://dgv.tcag.ca/dgv/app/variant?id=esv3578391) | CNV | Loss | [25503493](http://www.ncbi.nlm.nih.gov/entrez/query.fcgi?db=PubMed&cmd=search&Dopt=b&term=25503493) |

| Table S6. Single nucleotide polymorphisms (SNPs) of CTSC, IL10, IL8, and IL18. | | | |
| --- | --- | --- | --- |
| **SNP ID** | **Position** | **Sequence Context** | **Type** |
| **CTSC** | | | |
| rs104894207 | chr11：88,300,532(-) | AAACC(A/T)AGGTA | reference, missense |
| rs104894208 | chr11：88,296,165(-) | CCCTC(A/G)GGAGG | reference, missense |
| rs104894210 | chr11：88,337,557(-) | CACCT(C/G)GGTCT | upstream-variant-2KB, reference, missense |
| rs104894211 | chr11：88,294,358(-) | CCACT(A/G)TGTAG | reference, missense |
| rs104894214 | chr11：88,294,497(-) | AAGGC(A/G)GCTTC | Missense |
| **IL8** | | | |
| rs1000003014 | chr4：73,739,370(+) | ACAGA(A/T)TCCAA | upstream-variant-2KB |
| rs1000022557 | chr4：73,743,126(+) | ATTTA(A/T)GTATT | utr-variant-3-prime |
| rs1000275946 | chr4：73,739,885(+) | GACCA(A/G)ACTCT | upstream-variant-2KB |
| rs1000557302 | chr4：73,740,928(+) | TAGAT(A/T)TTCTG | intron-variant |
| rs1000965738 | chr4：73,740,637(+) | GAAGG(A/G)ACCAT | utr-variant-5-prime |
| **IL18** | | | |
| rs1000120984 5 | chr11：112,145,765(+) | AAAAG(A/G)AAAAA | intron-variant |
| rs1000157208 5 | chr11：12,151,016(+) | TTAAT(G/T)TGCCA | intron-variant |
| rs1000352535 5 | chr11：112,160,072(+) | TCATT(C/T)TTAAC | intron-variant |
| rs1000372405 5 | chr11：112,144,531(+) | TGTAA(A/G)CCACC | intron-variant |
| rs1000722644 5 | chr11：112,144,350(+) | GCCTC(A/T)GCCTC | intron-variant |
| **IL10** | | | |
| rs1000660306 5 | 206,771,802(+) | TTAGC(C/T)TGGAA | intron-variant |
| rs1000968106 5 | 206,773,654(+) | AGTTG(C/G)GTTGC | upstream-variant-2KB |
| rs1001055509 5 | 206,767,177(+) | GGAGG(C/T)GGAGG | downstream-variant-500B |
| rs1001440172 5 | 206,773,941(+) | CCTGT(C/T)CTCCT | upstream-variant-2KB |
| rs1001662384 5 | 206,770,319(+) | CACCA(A/G)CTGAT | intron-variant |
